# [(1,2,5,6-η)-Cyclo­octa-1,5-diene]bis­(1-methyl-3-propylimidazol-2-yl­idene-κ*C*)iridium(I) tetra­fluorido­borate

**DOI:** 10.1107/S2414314626001896

**Published:** 2026-02-24

**Authors:** Benedikt N. Kienle, Michael Gau, Daniel R. Albert, Edward Rajaseelan

**Affiliations:** a Lancaster Country Day School. 725 Hamilton Road, Lancaster, PA 17603, USA; b Department of Chemistry, University of Pennsylvania, Philadelphia, PA 19104, USA; c Department of Chemistry Millersville University,Millersville PA 17551 USA; Vienna University of Technology, Austria

**Keywords:** crystal structure, bis N-heterocyclic carbenes, iridium, complex salt

## Abstract

In the title complex [Ir(C_8_H_12_)(C_7_H_12_N_2_)_2_]BF_4_, the central Ir^I^ atom of the cationic complex has a distorted square-planar coordination environment, formed by a bidentate cyclo­octa-1,5-diene (COD) ligand, and two N-heterocyclic carbene ligands. Non-classical hydrogen-bonding inter­actions between the [BF_4_]^−^ anion and the N-heterocyclic carbenes on three distinct cationic iridium(I) complexes serve to establish the orientation of the [BF_4_]^−^ anion in the extended structure.

## Abstract 2

The Ir^I^ atom is coordinated by a bidentate cyclo­octa-1,5-diene (COD) ligand and two N-heterocyclic carbene ligands, leading to a distorted square-planar coordination environment.

## Graphical abstract

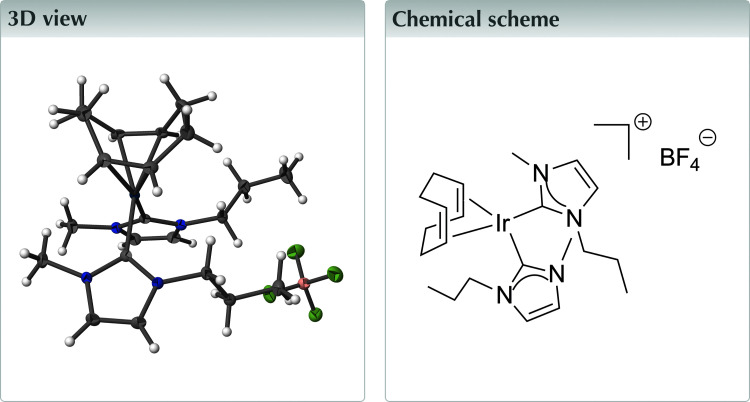

## Structure description

N-heterocyclic carbenes (NHCs) have emerged as excellent alternative ligands for phosphines to synthesize active metal complexes in homogeneous catalysis (Cazin, 2013 ▸; de Frémont *et al.*, 2009 ▸; Diez-González *et al.*, 2009 ▸; Rovis & Nolan, 2013 ▸; Ruff *et al.*, 2016 ▸; Zuo *et al.*, 2014 ▸). The use of these complexes as catalysts for the transfer hydrogenation of several unsaturated substrates has also been studied and reported (Albrecht *et al.*, 2002 ▸; Gnanamgari *et al.*, 2007 ▸; Hillier *et al.*, 2001 ▸). The NHC ligands can be tuned sterically and electronically by having different substituents (wing tips) on the nitro­gen atoms (Gusev, 2009 ▸). Though many imidazole-based NHC iridium complexes have been synthesized and structurally characterized (Herrmann *et al.*, 2006 ▸; Wang & Lin 1998 ▸; Chianese *et al.*, 2004 ▸), fewer structures of complexes with smaller wing-tip substituents have been reported. We continue to synthesize new imidazole and triazole-based NHC complexes of rhodium and iridium to study the effect of different substituents on the NHCs and the other ligands coordinating to the metal in transfer hydrogenation reactions (Nichol *et al.*, 2009 ▸, 2010 ▸, 2011 ▸, 2012 ▸; Idrees *et al.*, 2017*a* ▸,*b* ▸; Rood *et al.*, 2021 ▸; Rushlow *et al.*, 2021 ▸; Newman *et al.*, 2021 ▸; Castaldi *et al.*, 2021 ▸; Maynard *et al.*, 2023 ▸; Lerch *et al.*, 2024 ▸, 2025 ▸). Here we report the structure of an iridium complex with two identical imidazole-based monodentate carbene ligands.

**Figure 1:**
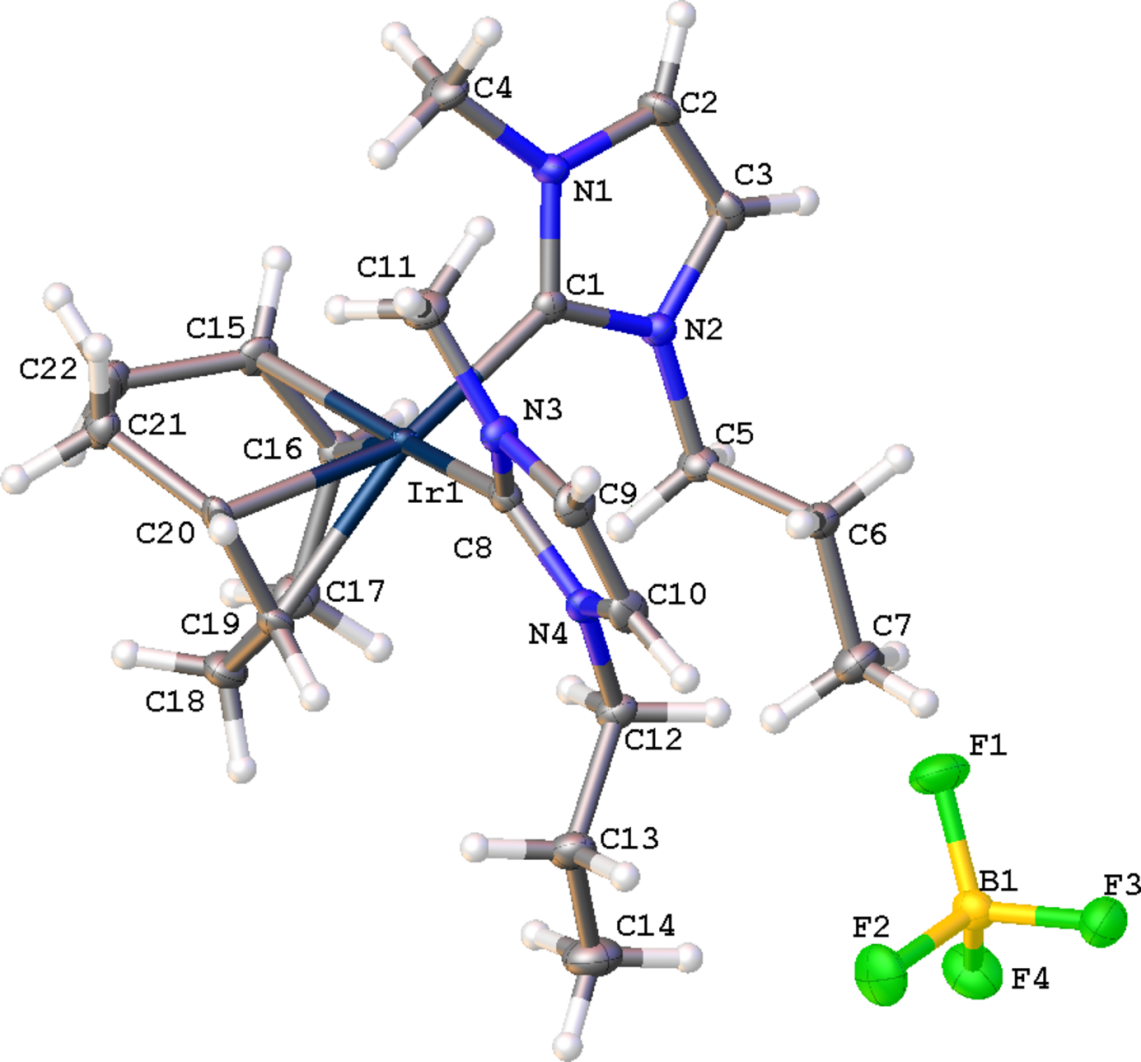
Mol­ecular structure of the title compound (**3**) with displacement ellipsoids drawn at the 50% probability level.

**Figure 2:**
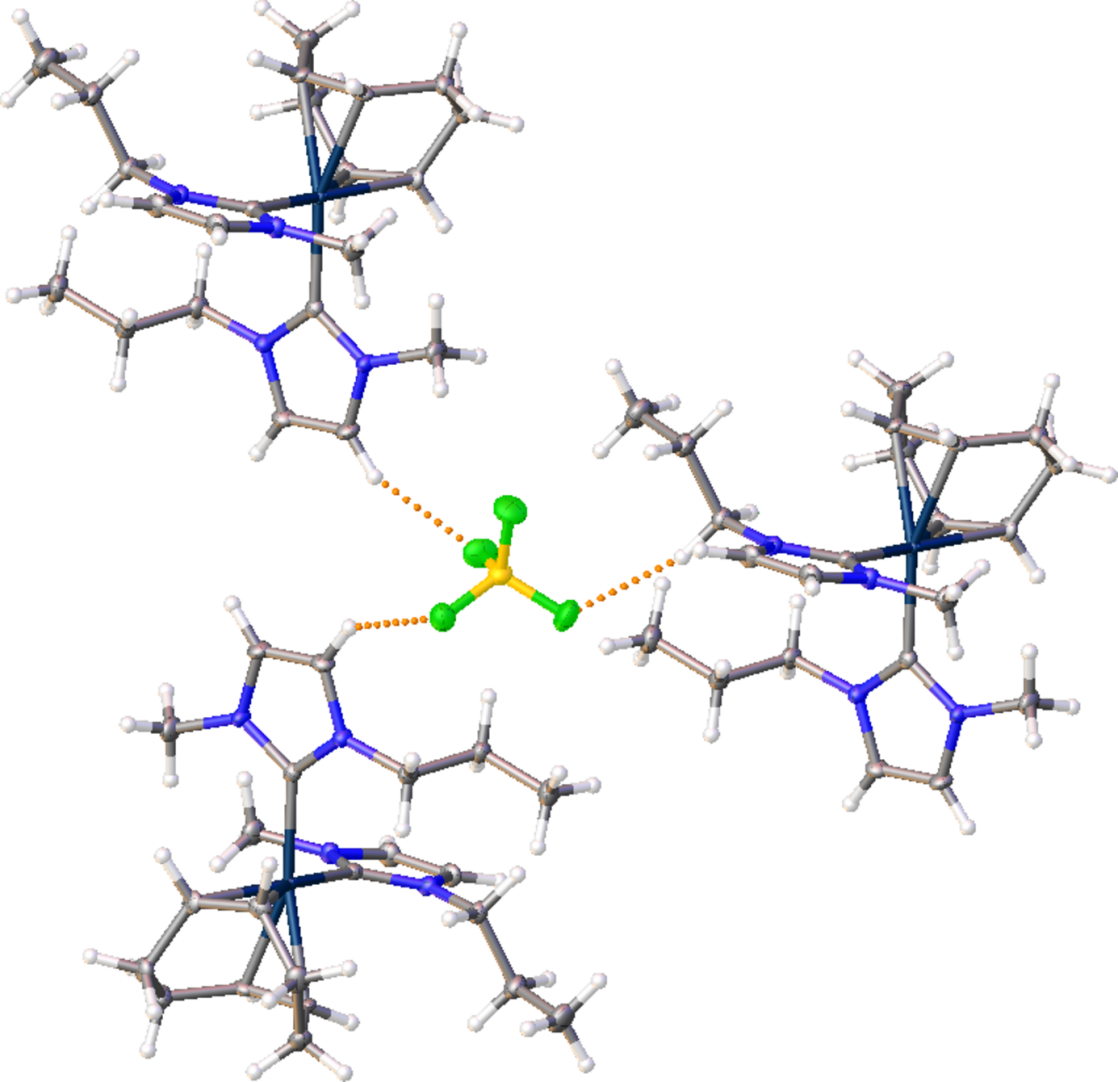
The title compound (**3**) showing the C—H⋯F hydrogen bonds (dotted orange lines) accepted by one [BF_4_]^−^ anion and the NHCs of three distinct iridium cations.

**Figure 3:**
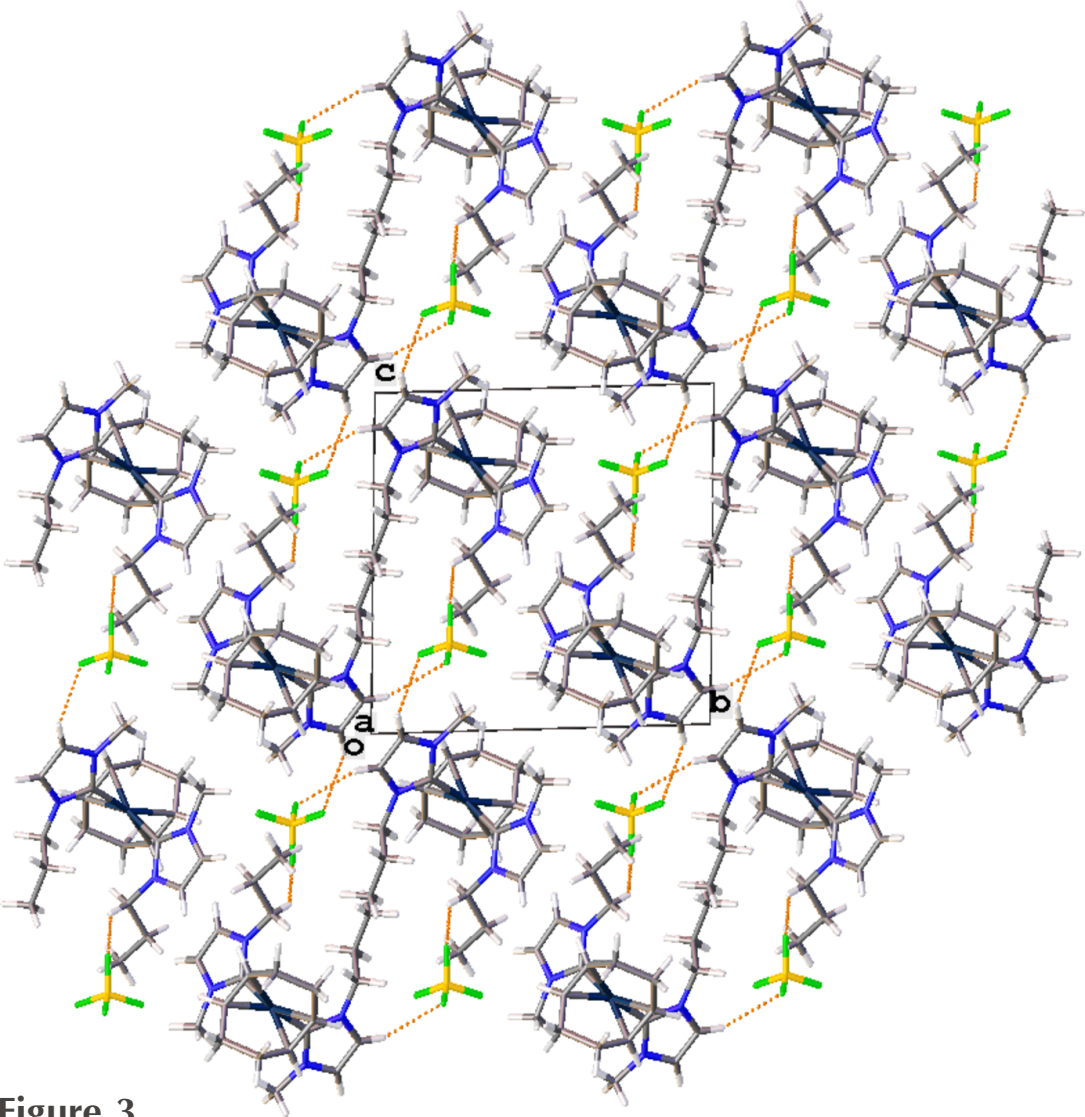
Packing diagram of the title compound shown along [100] with hydrogen-bonding inter­actions shown as dotted orange lines.

The mol­ecular structure of the title complex, [Ir(C_8_H_12_)(C_7_H_12_N_2_)][BF_4_], (**3**), comprises an Ir^I^ cation complex and a tetra­fluorido­borate counter-anion, illustrated in Fig. 1 ▸. No solvent mol­ecules are present in the crystal structure. The coordination environment of the central Ir^I^ atom of the cationic complex is distorted square-planar, defined by a bidentate cyclo­octa-1,5-diene (COD) ligand, and two NHC ligands. The carbene atoms, C1 and C8, deviate from the expected *sp*^2^ hybridization in that the N1—C1—N2 and the N4—C8—N3 bond angles in the imidazole-based carbenes are 103.9 (2) and 104.1 (2)°, respectively. Other selected bond lengths and angles in the structure are: Ir1—C1(NHC) 2.052 (2) Å, Ir1—C8(NHC) 2.052 (2) Å, and C1—Ir1—C8 is 94.62 (9)°. Non-classical C—H⋯F hydrogen-bonding inter­actions between the NHCs of the iridium cation and the tetra­fluorido­borate anion are summarized in Table 1 ▸. Notably, each [BF_4_]^−^ anion inter­acts with three separate cations as shown in Fig. 2 ▸. The crystal packing diagram of the complex is shown in Fig. 3 ▸, with the stabilizing H⋯F inter­actions shown as dotted orange lines. Two of the hydrogen-bonding inter­actions are with C—H groups of the NHC ring with the third inter­action occurring with the propyl wing tip of the NHC.

## Synthesis and crystallization

**Figure 4:**
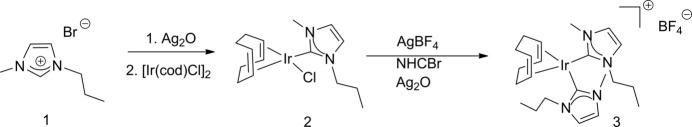
Reaction scheme for the synthesis of the title compound (**3**).

The synthesis scheme is shown in Fig. 4 ▸. All compounds used in the syntheses were obtained from Sigma-Aldrich and Strem and used as received; all syntheses were performed under a nitro­gen atmosphere. NMR spectra were recorded at room temperature in CDCl_3_ on a 400 MHz (operating at 100 MHz for ^13^C) Varian spectrometer and referenced to the residual solvent peak (δ in p.p.m.).

**1-Methyl-3-propylimidazolium bromide** (**1**) was synthesized by refluxing 1-methyl imidazole and 1-bromo­propane in toluene for 48 h under nitro­gen.

**[(1,2,5,6-η)-Cyclo­octa-1,5-diene](1-methyl-3-propylimida­zol-2-yl­idene)chlorido­iridium (2):** Imidazolium bromide (**1**) (0.061 g, 0.298 mmol) and Ag_2_O (0.034 g, 0.149 mmol) were stirred at room temperature in the dark for 1 h in CH_2_Cl_2_ (10 ml). The mixture was then filtered through Celite into [Ir(cod)Cl]_2_ (0.100 g, 0.149 mmol), and stirred again in the dark for 1.5 h. The resulting solution was filtered through Celite and the solvent was removed under reduced pressure in a rotary evaporator. The yellow solid product (**2**) was dried under vacuum. Yield: 0.130 g (95%). ^1^H NMR: δ 6.82 (*s*, 1H, N—C_4_H), 6.80 (*s*, 1 H, N—C_5_H), 4.57 (*s*, 3H, N—CH_3_), 4.37 (*m*, 2 H, CH of COD), 4.29 (*m*, 2H, CH of COD), 4.09 (*t*, 2H, N—CH_2_ of prop­yl), 1.97 (*m*, 2 H, CH_2_ of prop­yl), 1.85–1.60 (*m*, 8H, CH_2_ of COD), 1.01 (*t*, 3H, CH_3_ of prop­yl). ^13^C NMR: δ 180.11 (Ir—C), 121.59 (N—C_4_H), 119.84 (N—C_5_H), 84.16, 84.06 (CH of COD), 51.16 (N—CH_3_), 37.46 (N—CH_2_ of Pr), 33.76,33.39,29.76,29.40 (CH_2_ of COD), 24.25 (CH­_2_ of prop­yl), 11.40 (CH­_3_ of prop­yl).

**[(1,2,5,6-η)-Cyclo­octa-1,5-diene]bis­(1-methyl-3-propyl-imid­a­zol-2-yl­idene)iridium(I) tetra­fluorido­borate (3):** Imidazolium bromide (**1**) (0.055 g, 0.269 mmol) and Ag_2_O (0.031 g, 0.135 mmol) were stirred at room temperature in the dark for 1 h in CH_2_Cl_2_ (10 ml). The mixture was then filtered through Celite into a flask containing 0.124 g (0.269 mmol) of (**2**), in 10 ml of CH_2_Cl_2_. The solution was stirred in the dark for 1.5 h. The resulting mixture was filtered through Celite and the solvent was removed under reduced pressure. The rust-orange solid product (**3**) was dried under vacuum. Compound (**3**) was crystallized in the form of orange blocks suitable for data collection by slow diffusion of pentane into a CH_2_Cl­_2_ solution. Yield: 0.170 g (99%). ^1^H NMR: δ 7.10–7.00 (*m*, 4H, N—C_4_H, N—C_5_H), 4.39, 4.37 (*m*, 6H, N—CH_3_), 4.22–4.15 (*m*, 4H, CH of COD), 3.96 (*m*, 4H, N—CH_2_ of prop­yl), 3.82 (*m*, 2 H, CH_2_ of COD), 3.76 (2.04 (*m*, 4H, CH_2_ of prop­yl), 1.94–1.87 (*m*, 8H, CH_2_ of COD), 1.02 (*m*, 6H, CH_3_ of prop­yl). ^13^C NMR: δ 176.19, 176.16 (Ir—C), 123.56, 123.34 (N—C_4_H), 120.78, 120.39 (N—C_5_H), 76.09, 75.27, 74.45 (CH of COD), 52.05,51.37 (N—CH_3_), 38.12, 37.98 (N—CH_2_ of prop­yl), 32.99, 31.40, 31.31, 29.69 (CH_2_ of COD), 23.72, 23.50 (CH_2_ of prop­yl), 11.38, 11.29 (CH_3_ of prop­yl).

## Refinement

Crystal data, data collection and structure refinement details are summarized in Table 2 ▸.

## Supplementary Material

Crystal structure: contains datablock(s) I. DOI: 10.1107/S2414314626001896/wm4245sup1.cif

Structure factors: contains datablock(s) I. DOI: 10.1107/S2414314626001896/wm4245Isup2.hkl

CCDC reference: 2532047

Additional supporting information:  crystallographic information; 3D view; checkCIF report

## Figures and Tables

**Table 1:** Hydrogen-bond geometry (Å, °)

*D*—H⋯*A*	*D*—H	H⋯*A*	*D*⋯*A*	*D*—H⋯*A*
C2—H2⋯F4^i^	0.95	2.53 (1)	3.380 (3)	150 (1)
C3—H3⋯F3^ii^	0.95	2.53 (1)	3.336 (3)	143 (1)
C12—H12*b*⋯F1	0.99	2.51 (1)	3.354 (3)	144 (1)

Symmetry codes: (i) 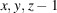; (ii) 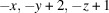.

**Table 2:** Experimental details

Crystal data	
Chemical formula	[Ir(C_8_H_12_)(C_7_H_12_N_2_)_2_]BF_4_
*M*_r_	635.59
Crystal system, space group	Triclinic, *P* 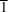
Temperature (K)	100
*a*, *b*, *c* (Å)	8.0252 (2), 12.1221 (3), 12.2566 (3)
α, β, γ (°)	87.486 (2), 83.233 (2), 87.913 (2)
*V* (Å^3^)	1182.32 (5)
*Z*	2
Radiation type	Mo *K*α
μ (mm^−1^)	5.71
Crystal size (mm)	0.13 × 0.08 × 0.03

Data collection	
Diffractometer	Rigaku XtaLAB Synergy-S
Absorption correction	Multi-scan (*CrysAlis PRO*; Rigaku OD, 2025 ▸)
*T*_min_, *T*_max_	0.582, 1.000
No. of measured, independent and observed [*I* ≥ 2u(*I*)] reflections	36081, 5869, 5452
*R*_int_	0.050
(sin θ/λ)_max_ (Å^−1^)	0.667

Refinement	
*R*[*F*^2^ > 2σ(*F*^2^)], *wR*(*F*^2^), *S*	0.020, 0.042, 1.02
No. of reflections	5869
No. of parameters	293
H-atom treatment	H-atom parameters constrained
Δρ_max_, Δρ_min_ (e Å^−3^)	1.32, −0.89

Computer programs: *CrysAlis PRO* (Rigaku OD, 2025 ▸), *SHELXT* (Sheldrick, 2015 ▸), *OLEX2.refine* (Bourhis *et al.*, 2015 ▸), *OLEX2* (Dolomanov *et al.*, 2009 ▸) and *publCIF* (Westrip, 2010 ▸).

## full crystallographic data

### [(1,2,5,6-η)-Cycloocta-1,5-diene]bis(1-methyl-3-propylimidazol-2-ylidene-κC)iridium(I) tetrafluoridoborate Crystal data

**Table:**

[Ir(C_8_H_12_)(C_7_H_12_N_2_)_2_]·BF_4_	*Z* = 2
*M**_r_* = 635.59	*F*(000) = 626.581
Triclinic, *P*1	*D*_x_ = 1.785 Mg m^−^^3^
*a* = 8.0252 (2) Å	Mo *K*α radiation, λ = 0.71073 Å
*b* = 12.1221 (3) Å	Cell parameters from 26079 reflections
*c* = 12.2566 (3) Å	θ = 3.6–28.3°
α = 87.486 (2)°	µ = 5.71 mm^−^^1^
β = 83.233 (2)°	*T* = 100 K
γ = 87.913 (2)°	Plate, orange
*V* = 1182.32 (5) Å^3^	0.13 × 0.08 × 0.03 mm

### [(1,2,5,6-η)-Cycloocta-1,5-diene]bis(1-methyl-3-propylimidazol-2-ylidene-κC)iridium(I) tetrafluoridoborate Data collection

**Table:**

Rigaku XtaLAB Synergy-S diffractometer	5869 independent reflections
Radiation source: micro-focus sealed X-ray tube, PhotonJet (Mo) X-ray Source	5452 reflections with *I*≥ 2u(*I*)
Mirror monochromator	*R*_int_ = 0.050
Detector resolution: 10.0 pixels mm^-1^	θ_max_ = 28.3°, θ_min_ = 3.2°
ω scans	*h* = −10→10
Absorption correction: multi-scan (CrysAlisPro; Rigaku OD, 2025)	*k* = −16→16
*T*_min_ = 0.582, *T*_max_ = 1.000	*l* = −16→16
36081 measured reflections	

### [(1,2,5,6-η)-Cycloocta-1,5-diene]bis(1-methyl-3-propylimidazol-2-ylidene-κC)iridium(I) tetrafluoridoborate Refinement

**Table:**

Refinement on *F*^2^	0 restraints
Least-squares matrix: full	56 constraints
*R*[*F*^2^ > 2σ(*F*^2^)] = 0.020	H-atom parameters constrained
*wR*(*F*^2^) = 0.042	*w* = 1/[σ^2^(*F*_o_^2^) + (0.016*P*)^2^ + 0.6194*P*] where *P* = (*F*_o_^2^ + 2*F*_c_^2^)/3
*S* = 1.02	(Δ/σ)_max_ = 0.0004
5869 reflections	Δρ_max_ = 1.32 e Å^−^^3^
293 parameters	Δρ_min_ = −0.89 e Å^−^^3^

### [(1,2,5,6-η)-Cycloocta-1,5-diene]bis(1-methyl-3-propylimidazol-2-ylidene-κC)iridium(I) tetrafluoridoborate Fractional atomic coordinates and isotropic or equivalent isotropic displacement parameters (Å^2^)

**Table:**

	*x*	*y*	*z*	*U*_iso_*/*U*_eq_	
Ir1	0.549050 (11)	0.715665 (7)	0.205112 (7)	0.01026 (3)	
N1	0.3239 (3)	0.81530 (16)	0.03638 (17)	0.0141 (4)	
N2	0.3191 (3)	0.92069 (16)	0.17287 (17)	0.0137 (4)	
N3	0.2859 (3)	0.53777 (16)	0.26516 (17)	0.0144 (4)	
N4	0.3082 (3)	0.63481 (16)	0.40447 (17)	0.0138 (4)	
C1	0.3824 (3)	0.82161 (19)	0.1357 (2)	0.0124 (5)	
C2	0.2255 (3)	0.9081 (2)	0.0126 (2)	0.0174 (5)	
H2	0.1709 (3)	0.9222 (2)	−0.0515 (2)	0.0209 (6)*	
C3	0.2230 (3)	0.9741 (2)	0.0983 (2)	0.0165 (5)	
H3	0.1663 (3)	1.0440 (2)	0.1062 (2)	0.0198 (6)*	
C4	0.3724 (4)	0.7291 (2)	−0.0423 (2)	0.0209 (6)	
H4a	0.442 (2)	0.7607 (4)	−0.1062 (7)	0.0313 (8)*	
H4b	0.436 (2)	0.6698 (8)	−0.0072 (5)	0.0313 (8)*	
H4c	0.2714 (4)	0.6993 (11)	−0.0662 (12)	0.0313 (8)*	
C5	0.3494 (3)	0.9661 (2)	0.2778 (2)	0.0165 (5)	
H5a	0.4475 (3)	0.9267 (2)	0.3050 (2)	0.0197 (6)*	
H5b	0.3764 (3)	1.0451 (2)	0.2655 (2)	0.0197 (6)*	
C6	0.1984 (3)	0.9556 (2)	0.3646 (2)	0.0175 (5)	
H6a	0.1013 (3)	0.9977 (2)	0.3391 (2)	0.0210 (6)*	
H6b	0.1683 (3)	0.8770 (2)	0.3750 (2)	0.0210 (6)*	
C7	0.2352 (4)	0.9989 (2)	0.4742 (2)	0.0234 (6)	
H7a	0.242 (2)	1.0795 (3)	0.4679 (5)	0.0351 (9)*	
H7b	0.1450 (13)	0.9784 (14)	0.5318 (4)	0.0351 (9)*	
H7c	0.3422 (12)	0.9666 (12)	0.4932 (8)	0.0351 (9)*	
C8	0.3667 (3)	0.62583 (19)	0.2968 (2)	0.0139 (5)	
C9	0.1781 (3)	0.4938 (2)	0.3517 (2)	0.0188 (5)	
H9	0.1081 (3)	0.4326 (2)	0.3498 (2)	0.0225 (6)*	
C10	0.1919 (3)	0.5546 (2)	0.4383 (2)	0.0177 (5)	
H10	0.1330 (3)	0.5448 (2)	0.5098 (2)	0.0213 (6)*	
C11	0.3069 (3)	0.4958 (2)	0.1546 (2)	0.0204 (5)	
H11a	0.2387 (18)	0.5413 (10)	0.1076 (4)	0.0306 (8)*	
H11b	0.4254 (5)	0.4985 (14)	0.1243 (6)	0.0306 (8)*	
H11c	0.271 (2)	0.4192 (5)	0.1575 (3)	0.0306 (8)*	
C12	0.3632 (3)	0.71348 (19)	0.4791 (2)	0.0157 (5)	
H12a	0.4511 (3)	0.75974 (19)	0.4386 (2)	0.0188 (6)*	
H12b	0.2672 (3)	0.76281 (19)	0.5054 (2)	0.0188 (6)*	
C13	0.4323 (4)	0.6551 (2)	0.5773 (2)	0.0217 (6)	
H13a	0.3412 (4)	0.6151 (2)	0.6222 (2)	0.0260 (7)*	
H13b	0.5206 (4)	0.6002 (2)	0.5510 (2)	0.0260 (7)*	
C14	0.5052 (4)	0.7364 (3)	0.6477 (2)	0.0307 (7)	
H14a	0.6003 (17)	0.7725 (13)	0.6047 (6)	0.0461 (10)*	
H14b	0.4189 (9)	0.7923 (10)	0.6717 (15)	0.0461 (10)*	
H14c	0.544 (2)	0.6971 (4)	0.7122 (9)	0.0461 (10)*	
C15	0.7544 (3)	0.7682 (2)	0.0800 (2)	0.0160 (5)	
H15	0.7161 (3)	0.8000 (2)	0.0101 (2)	0.0192 (6)*	
C16	0.7361 (3)	0.8405 (2)	0.1663 (2)	0.0167 (5)	
H16	0.6881 (3)	0.9150 (2)	0.1465 (2)	0.0200 (6)*	
C17	0.8466 (3)	0.8414 (2)	0.2566 (2)	0.0218 (6)	
H17a	0.9543 (3)	0.8748 (2)	0.2271 (2)	0.0261 (7)*	
H17b	0.7922 (3)	0.8889 (2)	0.3151 (2)	0.0261 (7)*	
C18	0.8840 (3)	0.7260 (2)	0.3081 (2)	0.0217 (6)	
H18a	0.9871 (3)	0.6938 (2)	0.2677 (2)	0.0261 (7)*	
H18b	0.9050 (3)	0.7337 (2)	0.3853 (2)	0.0261 (7)*	
C19	0.7406 (3)	0.6479 (2)	0.3053 (2)	0.0158 (5)	
H19	0.6938 (3)	0.6179 (2)	0.3795 (2)	0.0189 (6)*	
C20	0.7253 (3)	0.5773 (2)	0.2198 (2)	0.0152 (5)	
H20	0.6704 (3)	0.5065 (2)	0.2450 (2)	0.0183 (6)*	
C21	0.8430 (3)	0.5686 (2)	0.1151 (2)	0.0177 (5)	
H21a	0.9445 (3)	0.5247 (2)	0.1308 (2)	0.0213 (6)*	
H21b	0.7874 (3)	0.5285 (2)	0.0614 (2)	0.0213 (6)*	
C22	0.8961 (3)	0.6809 (2)	0.0635 (2)	0.0187 (5)	
H22a	0.9303 (3)	0.6735 (2)	−0.0162 (2)	0.0224 (6)*	
H22b	0.9943 (3)	0.7052 (2)	0.0970 (2)	0.0224 (6)*	
F1	−0.0053 (2)	0.77004 (14)	0.62832 (14)	0.0317 (4)	
F2	0.0743 (2)	0.66988 (13)	0.77507 (15)	0.0325 (4)	
F3	−0.1646 (2)	0.77746 (13)	0.79418 (14)	0.0270 (4)	
F4	0.0893 (2)	0.85740 (13)	0.76949 (15)	0.0312 (4)	
B1	−0.0009 (4)	0.7694 (2)	0.7411 (3)	0.0189 (6)	

### [(1,2,5,6-η)-Cycloocta-1,5-diene]bis(1-methyl-3-propylimidazol-2-ylidene-κC)iridium(I) tetrafluoridoborate Atomic displacement parameters (Å^2^)

**Table:**

	*U*^11^	*U*^22^	*U*^33^	*U*^12^	*U*^13^	*U*^23^
Ir1	0.01091 (5)	0.01099 (5)	0.00872 (5)	−0.00063 (3)	−0.00122 (3)	0.00178 (3)
N1	0.0173 (10)	0.0140 (10)	0.0115 (10)	0.0022 (8)	−0.0040 (8)	−0.0007 (8)
N2	0.0163 (10)	0.0121 (9)	0.0128 (10)	0.0013 (8)	−0.0026 (8)	0.0003 (8)
N3	0.0131 (10)	0.0158 (10)	0.0142 (11)	−0.0031 (8)	−0.0006 (8)	−0.0003 (8)
N4	0.0135 (10)	0.0154 (10)	0.0123 (10)	−0.0015 (8)	−0.0009 (8)	0.0030 (8)
C1	0.0128 (11)	0.0122 (11)	0.0117 (12)	−0.0021 (9)	0.0003 (9)	0.0016 (9)
C2	0.0201 (13)	0.0170 (12)	0.0153 (13)	0.0021 (10)	−0.0059 (10)	0.0045 (9)
C3	0.0177 (12)	0.0127 (11)	0.0187 (13)	0.0022 (10)	−0.0030 (10)	0.0047 (9)
C4	0.0283 (14)	0.0195 (12)	0.0156 (13)	0.0017 (11)	−0.0045 (11)	−0.0061 (10)
C5	0.0188 (12)	0.0158 (12)	0.0154 (13)	−0.0004 (10)	−0.0039 (10)	−0.0036 (9)
C6	0.0170 (12)	0.0210 (12)	0.0146 (13)	0.0009 (10)	−0.0021 (10)	−0.0018 (10)
C7	0.0271 (15)	0.0263 (14)	0.0165 (14)	−0.0004 (12)	0.0001 (11)	−0.0040 (11)
C8	0.0125 (11)	0.0145 (11)	0.0150 (12)	0.0013 (9)	−0.0030 (9)	0.0003 (9)
C9	0.0144 (12)	0.0202 (12)	0.0212 (14)	−0.0061 (10)	0.0011 (10)	0.0013 (10)
C10	0.0125 (12)	0.0216 (12)	0.0179 (13)	−0.0047 (10)	0.0026 (10)	0.0049 (10)
C11	0.0235 (14)	0.0205 (13)	0.0182 (14)	−0.0037 (11)	−0.0039 (11)	−0.0054 (10)
C12	0.0199 (13)	0.0145 (11)	0.0127 (12)	−0.0017 (10)	−0.0015 (10)	−0.0004 (9)
C13	0.0267 (14)	0.0240 (13)	0.0143 (13)	0.0012 (11)	−0.0032 (11)	0.0012 (10)
C14	0.0352 (17)	0.0383 (17)	0.0205 (15)	−0.0042 (14)	−0.0089 (13)	−0.0038 (12)
C15	0.0164 (12)	0.0173 (12)	0.0129 (12)	−0.0044 (10)	0.0031 (9)	0.0047 (9)
C16	0.0141 (12)	0.0145 (11)	0.0210 (14)	−0.0036 (10)	−0.0002 (10)	0.0028 (10)
C17	0.0189 (13)	0.0240 (13)	0.0230 (15)	−0.0072 (11)	−0.0020 (11)	−0.0039 (11)
C18	0.0171 (13)	0.0306 (14)	0.0185 (14)	−0.0003 (11)	−0.0061 (10)	−0.0009 (11)
C19	0.0151 (12)	0.0197 (12)	0.0125 (12)	0.0025 (10)	−0.0041 (9)	0.0039 (9)
C20	0.0134 (11)	0.0140 (11)	0.0173 (13)	0.0037 (9)	−0.0010 (9)	0.0056 (9)
C21	0.0210 (13)	0.0154 (12)	0.0160 (13)	0.0032 (10)	0.0000 (10)	−0.0003 (9)
C22	0.0171 (12)	0.0223 (13)	0.0152 (13)	−0.0021 (10)	0.0041 (10)	0.0010 (10)
F1	0.0382 (10)	0.0414 (10)	0.0159 (9)	−0.0028 (8)	−0.0038 (7)	−0.0016 (7)
F2	0.0374 (10)	0.0181 (8)	0.0413 (11)	0.0067 (7)	−0.0056 (8)	0.0030 (7)
F3	0.0254 (9)	0.0300 (8)	0.0252 (9)	0.0002 (7)	−0.0009 (7)	−0.0036 (7)
F4	0.0335 (9)	0.0217 (8)	0.0412 (11)	−0.0053 (7)	−0.0136 (8)	−0.0047 (7)
B1	0.0215 (15)	0.0158 (13)	0.0195 (16)	0.0017 (12)	−0.0041 (12)	0.0010 (11)

### [(1,2,5,6-η)-Cycloocta-1,5-diene]bis(1-methyl-3-propylimidazol-2-ylidene-κC)iridium(I) tetrafluoridoborate Geometric parameters (Å, º)

**Table:**

Ir1—C1	2.052 (2)	C11—H11a	0.9800
Ir1—C8	2.052 (2)	C11—H11b	0.9800
Ir1—C15	2.207 (2)	C11—H11c	0.9800
Ir1—C16	2.169 (2)	C12—H12a	0.9900
Ir1—C19	2.193 (2)	C12—H12b	0.9900
Ir1—C20	2.170 (2)	C12—C13	1.520 (4)
N1—C1	1.361 (3)	C13—H13a	0.9900
N1—C2	1.392 (3)	C13—H13b	0.9900
N1—C4	1.464 (3)	C13—C14	1.515 (4)
N2—C1	1.363 (3)	C14—H14a	0.9800
N2—C3	1.388 (3)	C14—H14b	0.9800
N2—C5	1.470 (3)	C14—H14c	0.9800
N3—C8	1.362 (3)	C15—H15	1.0000
N3—C9	1.387 (3)	C15—C16	1.395 (4)
N3—C11	1.458 (3)	C15—C22	1.527 (3)
N4—C8	1.356 (3)	C16—H16	1.0000
N4—C10	1.388 (3)	C16—C17	1.498 (4)
N4—C12	1.464 (3)	C17—H17a	0.9900
C2—H2	0.9500	C17—H17b	0.9900
C2—C3	1.346 (4)	C17—C18	1.543 (4)
C3—H3	0.9500	C18—H18a	0.9900
C4—H4a	0.9800	C18—H18b	0.9900
C4—H4b	0.9800	C18—C19	1.521 (4)
C4—H4c	0.9800	C19—H19	1.0000
C5—H5a	0.9900	C19—C20	1.401 (4)
C5—H5b	0.9900	C20—H20	1.0000
C5—C6	1.520 (3)	C20—C21	1.506 (3)
C6—H6a	0.9900	C21—H21a	0.9900
C6—H6b	0.9900	C21—H21b	0.9900
C6—C7	1.528 (4)	C21—C22	1.529 (3)
C7—H7a	0.9800	C22—H22a	0.9900
C7—H7b	0.9800	C22—H22b	0.9900
C7—H7c	0.9800	F1—B1	1.387 (4)
C9—H9	0.9500	F2—B1	1.398 (3)
C9—C10	1.336 (4)	F3—B1	1.398 (3)
C10—H10	0.9500	F4—B1	1.389 (3)
			
C8—Ir1—C1	94.62 (9)	H11c—C11—H11b	109.5
C15—Ir1—C1	90.86 (9)	H12a—C12—N4	109.28 (12)
C15—Ir1—C8	163.45 (9)	H12b—C12—N4	109.28 (13)
C16—Ir1—C1	87.71 (9)	H12b—C12—H12a	107.9
C16—Ir1—C8	158.51 (10)	C13—C12—N4	111.7 (2)
C16—Ir1—C15	37.16 (9)	C13—C12—H12a	109.28 (14)
C19—Ir1—C1	162.51 (9)	C13—C12—H12b	109.28 (14)
C19—Ir1—C8	91.27 (9)	H13a—C13—C12	109.38 (14)
C19—Ir1—C15	88.08 (9)	H13b—C13—C12	109.38 (14)
C19—Ir1—C16	80.93 (9)	H13b—C13—H13a	108.0
C20—Ir1—C1	158.75 (10)	C14—C13—C12	111.3 (2)
C20—Ir1—C8	89.27 (9)	C14—C13—H13a	109.38 (16)
C20—Ir1—C15	80.19 (9)	C14—C13—H13b	109.38 (16)
C20—Ir1—C16	96.27 (9)	H14a—C14—C13	109.5
C20—Ir1—C19	37.46 (10)	H14b—C14—C13	109.5
C2—N1—C1	111.5 (2)	H14b—C14—H14a	109.5
C4—N1—C1	125.1 (2)	H14c—C14—C13	109.5
C4—N1—C2	123.1 (2)	H14c—C14—H14a	109.5
C3—N2—C1	111.2 (2)	H14c—C14—H14b	109.5
C5—N2—C1	124.7 (2)	H15—C15—Ir1	114.29 (7)
C5—N2—C3	124.1 (2)	C16—C15—Ir1	69.96 (13)
C9—N3—C8	111.2 (2)	C16—C15—H15	114.29 (15)
C11—N3—C8	124.8 (2)	C22—C15—Ir1	112.56 (15)
C11—N3—C9	124.1 (2)	C22—C15—H15	114.29 (14)
C10—N4—C8	110.9 (2)	C22—C15—C16	123.6 (2)
C12—N4—C8	126.0 (2)	C15—C16—Ir1	72.87 (14)
C12—N4—C10	123.1 (2)	H16—C16—Ir1	113.77 (7)
N1—C1—Ir1	128.01 (17)	H16—C16—C15	113.77 (15)
N2—C1—Ir1	127.76 (18)	C17—C16—Ir1	109.60 (17)
N2—C1—N1	103.9 (2)	C17—C16—C15	125.5 (2)
H2—C2—N1	126.78 (14)	C17—C16—H16	113.77 (14)
C3—C2—N1	106.4 (2)	H17a—C17—C16	108.74 (14)
C3—C2—H2	126.78 (15)	H17b—C17—C16	108.74 (14)
C2—C3—N2	107.0 (2)	H17b—C17—H17a	107.6
H3—C3—N2	126.50 (13)	C18—C17—C16	114.0 (2)
H3—C3—C2	126.50 (15)	C18—C17—H17a	108.74 (14)
H4a—C4—N1	109.5	C18—C17—H17b	108.74 (15)
H4b—C4—N1	109.5	H18a—C18—C17	109.11 (14)
H4b—C4—H4a	109.5	H18b—C18—C17	109.11 (15)
H4c—C4—N1	109.5	H18b—C18—H18a	107.8
H4c—C4—H4a	109.5	C19—C18—C17	112.5 (2)
H4c—C4—H4b	109.5	C19—C18—H18a	109.11 (14)
H5a—C5—N2	109.17 (12)	C19—C18—H18b	109.11 (14)
H5b—C5—N2	109.17 (12)	C18—C19—Ir1	112.43 (16)
H5b—C5—H5a	107.9	H19—C19—Ir1	113.86 (6)
C6—C5—N2	112.2 (2)	H19—C19—C18	113.86 (14)
C6—C5—H5a	109.17 (14)	C20—C19—Ir1	70.35 (14)
C6—C5—H5b	109.17 (13)	C20—C19—C18	124.7 (2)
H6a—C6—C5	109.38 (14)	C20—C19—H19	113.86 (14)
H6b—C6—C5	109.38 (13)	C19—C20—Ir1	72.20 (14)
H6b—C6—H6a	108.0	H20—C20—Ir1	113.57 (6)
C7—C6—C5	111.3 (2)	H20—C20—C19	113.57 (14)
C7—C6—H6a	109.38 (14)	C21—C20—Ir1	110.01 (16)
C7—C6—H6b	109.38 (14)	C21—C20—C19	126.2 (2)
H7a—C7—C6	109.5	C21—C20—H20	113.57 (13)
H7b—C7—C6	109.5	H21a—C21—C20	108.94 (13)
H7b—C7—H7a	109.5	H21b—C21—C20	108.94 (14)
H7c—C7—C6	109.5	H21b—C21—H21a	107.8
H7c—C7—H7a	109.5	C22—C21—C20	113.2 (2)
H7c—C7—H7b	109.5	C22—C21—H21a	108.94 (14)
N3—C8—Ir1	127.60 (18)	C22—C21—H21b	108.94 (15)
N4—C8—Ir1	128.11 (18)	C21—C22—C15	111.8 (2)
N4—C8—N3	104.1 (2)	H22a—C22—C15	109.26 (14)
H9—C9—N3	126.73 (14)	H22a—C22—C21	109.26 (14)
C10—C9—N3	106.5 (2)	H22b—C22—C15	109.26 (14)
C10—C9—H9	126.73 (15)	H22b—C22—C21	109.26 (15)
C9—C10—N4	107.3 (2)	H22b—C22—H22a	107.9
H10—C10—N4	126.35 (14)	F2—B1—F1	109.0 (2)
H10—C10—C9	126.35 (15)	F3—B1—F1	109.5 (2)
H11a—C11—N3	109.5	F3—B1—F2	108.8 (2)
H11b—C11—N3	109.5	F4—B1—F1	110.8 (2)
H11b—C11—H11a	109.5	F4—B1—F2	109.6 (2)
H11c—C11—N3	109.5	F4—B1—F3	109.1 (2)
H11c—C11—H11a	109.5		
			
Ir1—C1—N1—C2	−174.0 (2)	N3—C9—C10—N4	−0.2 (2)
Ir1—C1—N1—C4	−0.6 (3)	N4—C8—N3—C9	0.6 (2)
Ir1—C1—N2—C3	173.9 (2)	N4—C8—N3—C11	179.58 (18)
Ir1—C1—N2—C5	−5.6 (2)	N4—C12—C13—C14	174.2 (2)
Ir1—C8—N3—C9	176.1 (2)	C1—N1—C2—C3	0.3 (2)
Ir1—C8—N3—C11	−4.9 (3)	C1—N2—C3—C2	−0.1 (2)
Ir1—C8—N4—C10	−176.2 (2)	C1—N2—C5—C6	−103.8 (2)
Ir1—C8—N4—C12	0.8 (3)	C2—C3—N2—C5	179.42 (19)
Ir1—C15—C16—C17	102.20 (15)	C3—N2—C5—C6	76.8 (2)
Ir1—C15—C22—C21	14.39 (19)	C3—C2—N1—C4	−173.1 (2)
Ir1—C16—C15—C22	−104.36 (13)	C8—N3—C9—C10	−0.2 (2)
Ir1—C16—C17—C18	36.38 (18)	C8—N4—C10—C9	0.6 (2)
Ir1—C19—C18—C17	9.70 (19)	C8—N4—C12—C13	−121.2 (3)
Ir1—C19—C20—C21	102.15 (14)	C9—C10—N4—C12	−176.5 (2)
Ir1—C20—C19—C18	−104.21 (14)	C10—N4—C12—C13	55.5 (2)
Ir1—C20—C21—C22	38.70 (19)	C10—C9—N3—C11	−179.2 (2)
N1—C1—N2—C3	0.3 (2)	C15—C16—C17—C18	−46.2 (3)
N1—C1—N2—C5	−179.22 (17)	C15—C22—C21—C20	−35.1 (3)
N1—C2—C3—N2	−0.2 (2)	C16—C15—C22—C21	94.6 (3)
N2—C1—N1—C2	−0.4 (2)	C16—C17—C18—C19	−30.8 (2)
N2—C1—N1—C4	172.96 (18)	C17—C16—C15—C22	−2.2 (3)
N2—C5—C6—C7	177.7 (2)	C17—C18—C19—C20	90.7 (2)
N3—C8—N4—C10	−0.7 (2)	C18—C19—C20—C21	−2.1 (3)
N3—C8—N4—C12	176.34 (17)	C19—C20—C21—C22	−43.4 (3)

### [(1,2,5,6-η)-Cycloocta-1,5-diene]bis(1-methyl-3-propylimidazol-2-ylidene-κC)iridium(I) tetrafluoridoborate Hydrogen-bond geometry (Å, º)

**Table:**

*D*—H···*A*	*D*—H	H···*A*	*D*···*A*	*D*—H···*A*
C2—H2···F4^i^	0.95	2.53 (1)	3.380 (3)	150 (1)
C3—H3···F3^ii^	0.95	2.53 (1)	3.336 (3)	143 (1)
C12—H12*b*···F1	0.99	2.51 (1)	3.354 (3)	144 (1)

Symmetry codes: (i) *x*, *y*, *z*−1; (ii) −*x*, −*y*+2, −*z*+1.
